# BP-METROLOGY: non-invasive continuous blood pressure monitoring to predict haemorrhagic transformation after endovascular thrombectomy

**DOI:** 10.1093/esj/aakag012

**Published:** 2026-03-23

**Authors:** Arthur Matthys, Laurine Bedoucha, Florent Di Meglio, Candice Sabben, Michael Obadia, Chloé Le Cossec, Julien Labreuche, Perrine Boursin, Mikael Mazighi, Lucas Di Meglio

**Affiliations:** Neuroglial Interactions in Cerebral Physiology and Pathologies, Center for Interdisciplinary Research in Biology, Collège de France, CNRS, INSERM, Labex Memolife, Université PSL, Paris, France; Medical Intensive Care Unit, Cochin Hospital, Assitance Publique Hôpitaux de Paris, APHP.Centre, Paris, France; Department of Neurology, Hôpital Lariboisière, Université Paris Cité, Assitance Publique - Hôpitaux de Paris, Paris, France; MINES ParisTech, Université PSL, Paris, France; Department of Neurology, Stroke Unit, Hôpital Fondation Adolphe de Rothschild, Paris, France; Department of Neurology, Stroke Unit, Hôpital Fondation Adolphe de Rothschild, Paris, France; Clinical Research Unit, Hôpital Fondation Adolphe de Rothschild, Paris, France; Department of Biostatistics, Centre Hospitalo-Universitaire de Lille, Lille, France; Clinical Research Unit, Hôpital Fondation Adolphe de Rothschild, Paris, France; Department of Neurology, Hôpital Lariboisière, Université Paris Cité, Assitance Publique - Hôpitaux de Paris, Paris, France; Department of Interventional Neuroradiology, Stroke Unit, Hôpital Fondation Adolphe de Rothschild, Paris, France; Optimisation Thérapeutique en Neuropharmacologie (INSERM UMR-S 1144), Université Paris Cité, Paris, France; Institut Universitaire de France, Paris, France; FHU NeuroVasc2030, Paris, France; Department of Neurology, Hôpital Lariboisière, Université Paris Cité, Assitance Publique - Hôpitaux de Paris, Paris, France; FHU NeuroVasc2030, Paris, France; Cardiovascular MArkers in Stressed COndiTions (INSERM UMR 942 MASCOT), Université Paris Cité, 2 rue Ambroise Paré, 75010 Paris, France

**Keywords:** acute ischaemic stroke, blood pressure monitoring, endovascular thrombectomy, haemorrhagic transformation

## Abstract

**Introduction:**

Haemorrhagic transformation (HT) seriously worsens functional outcome after endovascular therapy (EVT). Blood pressure (BP) variability may influence HT risk, but optimal monitoring strategies remain unclear. We aimed to determine whether continuous BP monitoring better identifies patients at risk of HT than standard intermittent measurements during the first 24 h post-EVT.

**Patients and methods:**

We conducted a single-centre prospective cohort study including adults with acute ischaemic stroke due to large-vessel occlusion treated with EVT. Non-invasive finger-cuff continuous BP and arm-cuff intermittent BP were recorded simultaneously for 24 h post-EVT. Haemorrhagic transformation on follow-up brain imaging at 24–36 h was the primary outcome. Blood pressure recordings were partitioned into three 8-h windows. Variability metrics (mean, maximum, range, SD, coefficient of variation and wavelet-based coefficient energies) were processed into logistic regression models adjusted for clinical covariates. Predictive performance was assessed using AUC-ROC.

**Results:**

Among 455 enrolled patients, 199 contributed data to the first 8-h window, in which HT occurred in 58 (29%). Continuous BP variability features, particularly maximum, range and wavelet energies capturing < 32-min fluctuations, were significantly associated with HT, whereas no parameter from intermittent monitoring showed such an association. A multivariable model using continuous data yielded an AUC-ROC of 0.62 (95% CI, 0.54–0.71) vs 0.48 (95% CI, 0.37–0.58) for intermittent data. Associations were not observed in later windows.

**Discussion and conclusion:**

Short-timescale BP variability captured by continuous monitoring in the first 8 h post-EVT is associated with increased HT risk, whereas intermittent monitoring fails to detect this signal and may miss opportunities for early risk stratification.

## Graphical Abstract

**Graphical Abstract:**
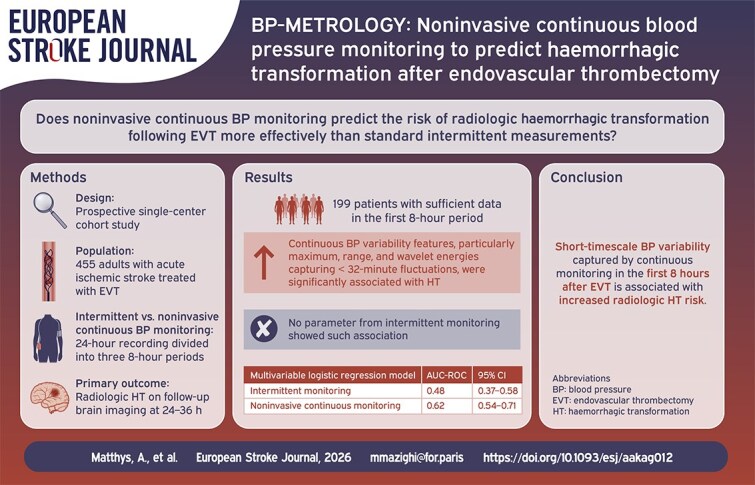

## Introduction

Endovascular therapy (EVT) has revolutionised the management of acute ischaemic stroke (AIS) by achieving high rates of recanalisation in proximal large-vessel occlusions.^1^ However, haemorrhagic transformation (HT) remains a critical complication and contributor to poor outcomes despite successful reperfusion.^2^ Blood pressure (BP) control following EVT has thus attracted considerable interest, as excessively high BP may increase the risk of intracranial bleeding, whereas low BP is more plausibly associated with hypoperfusion, infarct expansion and secondary tissue injury.^3^ Yet a single universal BP target post-EVT remains elusive,^4–6^ partly owing to interindividual differences in cerebral autoregulation and evolving haemodynamics during the early recovery phase.^7,8^

Because BP variability has been associated with HT and worse outcomes after successful EVT,^9–12^ there is growing interest in capturing continuous, high-resolution BP data rather than relying solely on standard intermittent arm-cuff measurements. Non-invasive arterial pressure monitoring, previously adopted in perioperative and intensive care settings,^13^ may offer enhanced temporal resolution and reveal short-term fluctuations critical to predicting HT risk. Although such devices have begun to show promise, their feasibility and usefulness in a specialised stroke care context remain to be fully established. Moreover, previous studies have inconsistently defined and often conflated short- and mid-term BP variability, while relying on invasive methods. Here, we used a non-invasive approach combined with wavelet decomposition, a signal processing technique akin to Fourier transform, well-suited to quantify variability across multiple timescales.^14^

In this prospective single-centre study, we examined whether continuous non-invasive BP monitoring identifies patients at higher risk of radiological HT more effectively than standard intermittent measurements. We focused on short-term BP variability features and their association with HT during the first 24 h after EVT.

## Patients and methods

### Study design and population

The BP-METROLOGY study was a single-centre, prospective cohort study conducted at the Fondation Adolphe de Rothschild Hospital from December 2018 to December 2023. We screened patients admitted with AIS due to occlusion of the internal carotid artery, proximal middle cerebral artery (M1 or M2 segment), vertebral artery or basilar artery. Inclusion criteria were: (i) age ≥ 18 years and (ii) decision to proceed with EVT. Exclusion criteria were haemorrhage prior to achieving reperfusion (eg, periprocedural intracranial haemorrhage) and pregnancy. The local institutional review board (IRB n° 2017-A01398-45) approved the study protocol, and all participants or their legally authorised representatives provided written informed consent. Stroke management—including IV thrombolysis administration, periprocedural antithrombotic use and initial BP targets—followed European Stroke Association guidelines.^15,16^

### Clinical data collection and primary outcome

Baseline demographics and clinical information were recorded, including: age, sex, vascular risk factors (hypertension, diabetes, smoking status, coronary disease, prior stroke or TIA), baseline antithrombotic therapy (antiplatelet or anticoagulant therapy), admission NIHSS score, time to reperfusion, intravenous thrombolytic therapy, periprocedural use of antithrombotic therapy (intra-arterial thrombolysis, heparin, antiplatelet therapy) and post-procedural use of antihypertensive drug.

The primary outcome was radiological HT, detected on systematic follow-up imaging (CT or MRI) at 24–36 h. HT was defined as any hyperdensity on CT or hypointensity on susceptibility-weighted images within the infarcted territory (from scattered small petechiae to intracerebral haemorrhage extending within and beyond infarcted brain tissue).^17^

### BP monitoring protocols

Each patient simultaneously underwent 2 BP monitoring protocols for 24 h post-EVT. Standard intermittent protocol consisted of brachial-cuff measurements every 15 min for 2 h, then 30 min for 6 h, then 60 min for the remaining 16 h. Systolic BP (SBP) and diastolic BP (DBP) were recorded. Mean BP (MBP) was calculated as SBP/3 + (2 × DBP/3). Concomitantly, non-invasive continuous BP monitoring was performed with a finger-cuff device (ClearSight, Edwards), providing measures for SBP, MBP and DBP. Continuous signals were averaged over 1-min intervals. Clinical teams remained blinded to continuous monitoring.

### Data analysis

Because previous studies have reported that early BP variability may be more strongly associated with HT than 24-h BP variability,^9^ we split the 24-h BP dataset into 3 consecutive 8-h periods. Data were preprocessed to unbiasedly remove artefacts and outliers. For each period, patients were included in the analysis if they had: (i) available data on primary outcome and clinical covariates for multivariable logistic regression (see below); (ii) at least 30% valid intermittent and continuous BP readings (ie, no more than 70% missing or artefact-contaminated data in each dataset) and (iii) a contiguous window of ≥128 min after outlier removal and imputation for gaps < 5 min, to ensure proper wavelet decomposition (see below). In this last case, imputation was performed by replacing missing or discarded values with the corresponding value of a Gaussian-filtered version of the signal. Comparisons between included and excluded patients were conducted to assess potential selection bias resulting from data availability constraints.

For each BP measure (SBP, DBP and MBP), we computed the following statistical features on both intermittent and continuous measurements, without imputation for missing values: mean, maximum, range, SD, coefficient of variation and proportions of measures above an absolute threshold (SBP > 185 mmHg, DBP > 110 mmHg, MBP > 130 mmHg). For continuous data, normalised coefficient energies were derived from wavelet decomposition, provided that a contiguous window of ≥ 128 min was available after imputation for gaps < 5 min. This minimum window length ensured reliable decomposition in 5 frequency bands, corresponding to oscillation periods of 1–2, 2–4, 4–8, 8–16 and 16–32 min.

We tested each *statistical* and *wavelet* feature’s association with the primary outcome using univariable and multivariable logistic regression. Multivariable logistic regression was adjusted for key clinical factors: categorised admission NIHSS scores (0–3, 4–14, 15–20, >20), admission MBP, time to reperfusion, IV thrombolysis and periprocedural antithrombotic drug administration. For each BP feature, computed OR shows the effect of 1 SD shift on the odds of radiological HT. We then evaluated predictive performance by training logistic regression models with all features from the first 8-h period for each dataset (continuous vs intermittent), applying a stratified 10-fold cross-validation. The area under the receiver operating characteristic curve (AUC-ROC) and the average precision (AP, area under the precision-recall curve) were computed with 95% CIs. We used paired *t*-tests on the results of the 10-folds to compare AUC-ROC and AP values between models. Statistical significance was set at *P* < .05 (2-tailed). In addition to the 3 prespecified 8-h windows, we performed analogous analyses using BP variability metrics aggregated over the full 24-h recording to assess whether associations persisted when a longer time interval was considered. Analyses were conducted using Python (version 3.11.8). Code is available upon reasonable request.

## Results

### Study population

**Figure 1:**
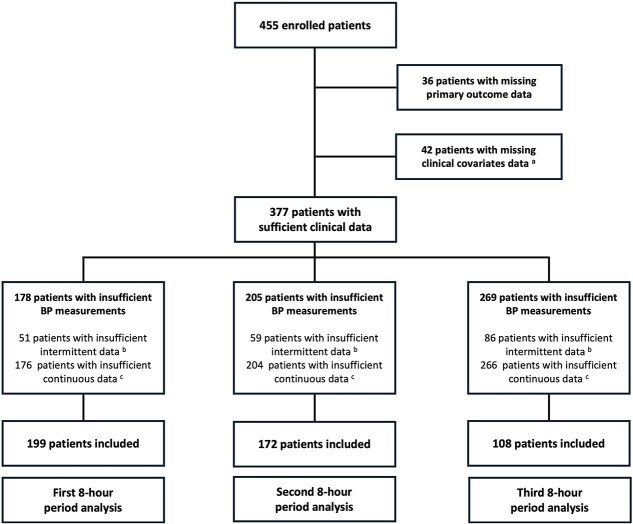
Flow chart. Patients were excluded if they had missing primary outcome data, missing clinical covariate data (^a^ admission NIHSS, admission mean blood pressure, time to reperfusion, intravenous thrombolysis, use of periprocedural antithrombotic therapies) or insufficient blood pressure measurements (^b^ < 30% of scheduled intermittent measurements; ^c^ < 30% of continuous measurements after artefact and outlier removal, or longest contiguous window < 128 min after imputation for gaps < 5 min) during the analysed periods.

Of the 455 patients initially enrolled in this study, 199 were included in the analysis for the first 8-h period post-EVT, 172 for the second and 108 for the third (Figure 1). Excluded patients had either no data on the primary outcome (*n* = 36), incomplete clinical information (*n* = 42) or incomplete BP data (*n* = 178 for the first 8-h period, 205 for the second and 269 for the third). Haemorrhagic transformation occurred in 58 (29%), 54 (31%) and 33 (31%) of the analysis subsets, respectively. Baseline characteristics and clinical information are described in Table 1, comparing patients with and without HT in each period population. Patients excluded from the analysis were significantly older than included patients for the first and second periods and had higher MBP values on admission for the first period (Table S1). No difference was observed in HT frequency between included and excluded patients in any period.

**Table 1:** Baseline characteristics and clinical information

	**Period 1 (0–8 h)**			**Period 2 (8–16 h)**			**Period 3 (16–24 h)**		
	**Overall (*n* = 199)**	**No HT (*n* = 141)**	**HT (*n* = 58)**	**Overall (*n* = 172)**	**No HT (*n* = 118)**	**HT (*n* = 54)**	**Overall (*n* = 108)**	**No HT (*n* = 75)**	**HT (*n* = 33)**
**Age (years), median [Q1, Q3]**	69.0 [55.0, 78.0]	69.0 [54.0, 78.0]	68.5 [59.0, 77.8]	69.0 [58.0, 79.0]	69.0 [55.2, 78.0]	68.5 [60.0, 80.0]	71.0 [59.0, 80.0]	72.0 [59.0, 79.0]	69.0 [60.0, 81.0]
**Sex, no (%)**									
**Female**	95 (48)	70 (50)	25 (43)	86 (50)	62 (53)	24 (44)	57 (53)	39 (52)	18 (55)
**Male**	104 (52)	71 (50)	33 (57)	86 (50)	56 (47)	30 (56)	51 (47)	36 (48)	15 (45)
**Hypertension, no (%)**	74 (37)	54 (38)	20 (34)	64 (37)	46 (39)	18 (33)	33 (31)	23 (31)	10 (30)
**Diabetes, no (%)**	163 (82)	119 (84)	44 (76)	140 (81)	99 (84)	41 (76)	90 (83)	67 (89)	23 (70)
**Smoking, no (%)**	165 (83)	113 (80)	52 (90)	142 (83)	95 (81)	47 (87)	92 (85)	61 (81)	31 (94)
**Coronary disease, no (%)**	187 (94)	131 (93)	56 (97)	162 (94)	112 (95)	50 (93)	103 (95)	72 (96)	31 (94)
**Prior stroke or TIA, no (%)**	177 (89)	125 (89)	52 (90)	153 (89)	105 (89)	48 (89)	93 (86)	64 (85)	29 (88)
**Any antithrombotic treatment, no (%)**	124 (62)	88 (62)	36 (62)	106 (62)	76 (64)	30 (56)	61 (56)	42 (56)	19 (58)
**Antiplatelet treatment, no (%)**	39 (20)	29 (21)	10 (17)	33 (19)	22 (19)	11 (20)	25 (23)	19 (25)	6 (18)
**Dual antiplatelet treatment, no (%)**	1 (1)	1 (1)		2 (1)	2 (2)		1 (1)	1 (1)	
**Anticoagulant treatment, no (%)**	37 (19)	25 (18)	12 (21)	32 (19)	20 (17)	12 (22)	23 (21)	15 (20)	8 (24)
**Admission MBP (mmHg)**	100 [90,114]	100 [91,114]	100 [89,116]	100 [91,114]	100 [91,113]	100 [90,117]	100 [92,112]	103 [93,113]	97 [86,107]
**NIHSS at admission, median [Q1, Q3]**	15.0 [11.0, 19.0]	15.0 [9.0, 18.0]	17.0 [14.0, 20.8]	15.0 [11.8, 18.0]	15.0 [10.0, 17.0]	17.5 [14.0, 22.5]	16.0 [13.0, 19.0]	15.0 [13.0, 18.0]	16.0 [13.0, 21.0]
**NIHSS (categorical), median [Q1, Q3]**									
**0–4**	17 (9)	16 (11)	1 (2)	11 (6)	10 (8)	1 (2)	3 (3)	3 (4)	
**5–15**	91 (46)	70 (50)	21 (36)	76 (44)	58 (49)	18 (33)	50 (46)	36 (48)	14 (42)
**16–20**	55 (28)	34 (24)	21 (36)	56 (33)	37 (31)	19 (35)	34 (31)	24 (32)	10 (30)
**≥ 21**	36 (18)	21 (15)	15 (26)	29 (17)	13 (11)	16 (30)	21 (19)	12 (16)	9 (27)
**IV thrombolysis, no (%)**	109 (55)	80 (57)	29 (50)	88 (51)	66 (56)	22 (41)	58 (54)	43 (57)	15 (45)
**Time to reperfusion (h), mean (SD)**	6.0 (5.3)	5.5 (4.1)	7.3 (7.5)	6.4 (7.5)	5.9 (7.3)	7.3 (7.8)	6.0 (5.5)	5.1 (2.0)	8.0 (9.2)
**mTICI score, no (%)**									
**0**	4 (2)	4 (3)		4 (2)	3 (3)	1 (2)	4 (4)	3 (4)	1 (3)
**1**				1 (1)		1 (2)			
**2A**	2 (1)	1 (1)	1 (2)	3 (2)	1 (1)	2 (4)	1 (1)	1 (1)	
**2B**	79 (40)	54 (38)	25 (43)	68 (40)	45 (38)	23 (43)	47 (44)	32 (43)	15 (45)
**3**	114 (57)	82 (58)	32 (55)	96 (56)	69 (58)	27 (50)	56 (52)	39 (52)	17 (52)
**Any periprocedural antithrombotic, no (%)**	84 (42)	54 (38)	30 (52)	73 (42)	43 (36)	30 (56)	50 (46)	29 (39)	21 (64)
**Intra-arterial thrombolysis, no (%)**	6 (3)	5 (4)	1 (2)	7 (4)	6 (5)	1 (2)	6 (6)	5 (7)	1 (3)
**Periprocedural heparin, no (%)**	72 (36)	44 (31)	28 (48)	61 (35)	33 (28)	28 (52)	40 (37)	22 (29)	18 (55)
**Periprocedural antiplatelet, no (%)**	9 (5)	7 (5)	2 (3)	6 (3)	5 (4)	1 (2)	6 (6)	4 (5)	2 (6)
**Post-procedural antihypertensive treatment, no (%)**	83 (42)	56 (40)	27 (47)	73 (42)	49 (42)	24 (44)	47 (44)	34 (45)	13 (39)

Abbreviations: HT = haemorrhagic transformation; MBP = mean blood pressure; mTICI = modified thrombolysis in cerebral infarction score.

### BP variability features and the risk of haemorrhagic transformation

**Figure 2:**
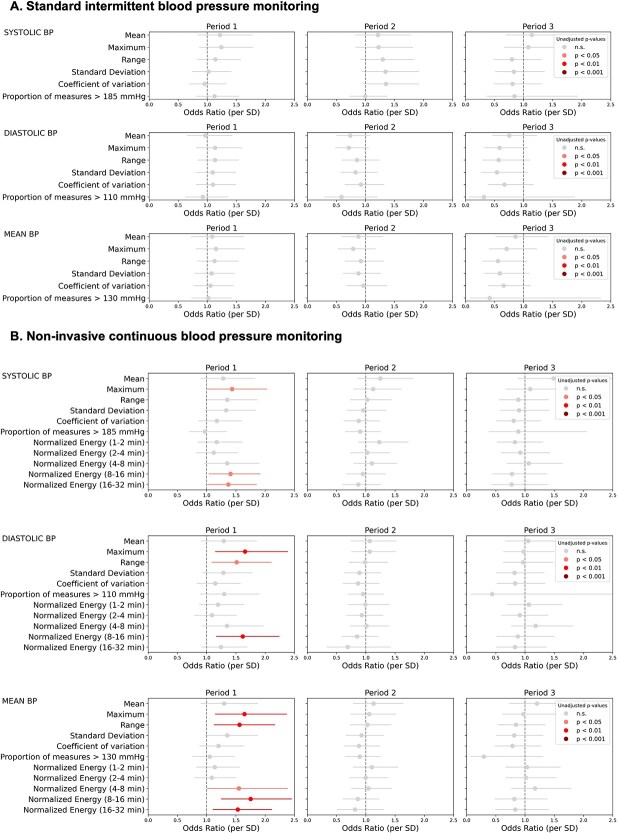
Multivariable logistic regression for association between BP variability features and post-EVT radiological HT. Effect of a 1 SD shift on the odds of radiological HT for each period, for features extracted from intermittent monitoring (A) and non-invasive continuous monitoring (B). Models were adjusted for admission NIHSS, admission mean blood pressure, time to reperfusion, intravenous thrombolysis and periprocedural antithrombotic administration. Units: Mean, maximum, range and SD in mmHg; coefficient of variation and proportion of measures above an absolute threshold in %; wavelet coefficients normalised energy in mmHg^2^/min. Abbreviations: BP = blood pressure; HT = haemorrhagic transformation.

No parameter derived from standard intermittent-cuff measurements was significantly associated with HT risk in any period. In contrast, continuous BP features in the first 8-h window—particularly maximum, range and wavelet coefficient energies capturing fluctuations in the 4- to 8-, 8- to 16- or 16- to 32-min frequency bands—were significantly associated with higher HT risk (Figure S1; Tables S2 and S3). When adjusted for admission NIHSS, admission MBP, time to reperfusion, IV thrombolysis and periprocedural use of antithrombotic therapies, these associations largely persisted (Figure 2; Tables S4 and S5). The second and third 8-h periods did not show consistent associations with HT in either monitoring method. Blood pressure variability metrics aggregated over the entire 24-h recording were also explored in the first subset of patients. In these analyses, no continuous BP variability metric remained significantly associated with the primary outcome after multivariable adjustment (Figure S2; Tables S6 and S7). This suggests that incorporating data from later time periods attenuated the observed associations, although reduced statistical power due to declining data availability over time cannot be formally excluded.

### Predictive modelling

**Figure 3:**
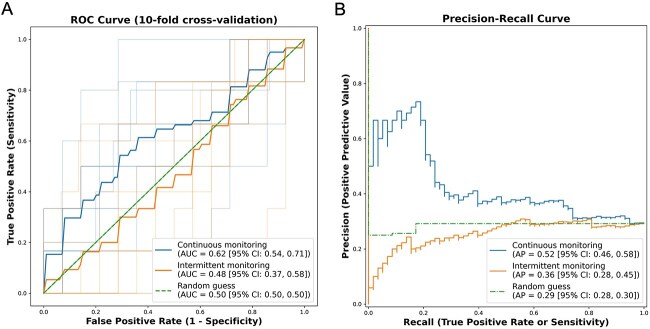
Performance of multivariable logistic regression models trained on intermittent or continuous BP dataset (first 8-h period) for the prediction of post-EVT radiological haemorrhagic transformation. ROC curve (A) and precision-recall curve (B) for multivariable logistic regression models trained on BP variability metrics displayed in Figure 1 for the first 8-h period, applying a stratified 10-fold cross-validation. The model trained on continuous data (blue) showed higher AUC-ROC (0.62 vs 0.48, *P* < .05) and AP (0.52 vs 0.36, *P* < .05) than the model trained on intermittent data (orange). Abbreviations: AUC = area under curve; AP = average precision; BP = blood pressure; OR = odds ratio; ROC = receiver operating characteristic.

The performances of continuous and standard intermittent BP monitoring protocols in predicting the risk of HT were compared by training a multivariable logistic regression model on all BP features derived from each protocol during the first 8-h period. The model trained on continuous measurements yielded an AUC-ROC of 0.62 (95% CI, 0.54–0.71) and an average precision of 0.52 (95% CI, 0.46–0.58), outperforming the model trained on intermittent measurements (AUC-ROC 0.48; 95% CI, 0.37–0.58, *P* < .05; average precision 0.36; 95% CI, 0.28–0.45; *P* < .05; Figure 3). When models were trained on BP variability metrics aggregated over 24 h, the model derived from continuous data did not significantly outperform the model derived from intermittent measurements (Figure S3). These findings highlight the potential of capturing early short-term BP variability to refine post-EVT stratification of HT risk.

## Discussion

In this prospective cohort of 455 patients with AIS treated by EVT, we found that short-term BP variability measured during the first 8 h post-reperfusion was significantly associated with the risk of radiological HT. Specifically, continuous BP features such as maximum, range, SD and wavelet coefficient energies corresponding to fluctuations < 32 min were independently linked to HT, even after adjustment for stroke severity, admission MBP, reperfusion delay, IV thrombolysis and periprocedural use of antithrombotic therapy. By contrast, no variability metric derived from standard intermittent-cuff measurements was associated with HT in any of the studied time windows. Moreover, predictive models trained on continuous BP data outperformed those based on intermittent data, with an AUC-ROC of 0.62 vs 0.48, respectively. These findings suggest that early short-timescale BP variability is a stronger and more actionable marker of haemorrhagic complications than mean BP levels or delayed instability. Nevertheless, predictive performance remained modest overall, indicating that BP information alone is not sufficient for precise individual-level prediction and that complementary clinical and imaging biomarkers will be necessary to improve risk stratification.

The temporal focus of our analyses was motivated by prior work suggesting that early BP variability may carry stronger prognostic value for haemorrhagic complications than BP variability assessed across longer intervals.^9^ Accordingly, our main analyses were performed on 3 consecutive 8-h periods, consistent with studies reporting stronger associations between early BP dynamics and haemorrhagic outcomes during the immediate post-reperfusion phase.

Our findings confirm previous observations that BP variability, rather than absolute BP levels, is a determinant of HT after AIS.^9–12^ They contrast however with several studies reporting associations between higher SBP variability derived from intermittent-cuff measurements and haemorrhagic outcomes after reperfusion therapies, including EVT.^9,10,12^ Discrepancies may arise from heterogeneous designs, endpoints (parenchymal haematomas or symptomatic intracranial haemorrhage) and BP variability metrics (eg, successive variation, time-rate of change). The absence of a significant association between intermittently derived BP variability and HT in our primary analyses should be interpreted in light of both methodological considerations and recent literature. To enable a direct head-to-head comparison between intermittent and continuous monitoring, we restricted analyses to patients with sufficient data available from both modalities within each time window, an approach that ensured comparability but inevitably resulted in a smaller analytic sample than in previous studies focusing solely on intermittent BP monitoring.^9,10,12^ In this context, continuous BP monitoring could represent a step towards more individualised risk assessment, where each patient contributes a rich temporal profile rather than a limited set of summary values. Interestingly, a recent individual patient-data meta-analysis including more than 2600 EVT-treated patients found that BP variability over the first 24 h, assessed using intermittent SBP SD and coefficient of variation, was associated with higher mortality and disability at 3 months but not with symptomatic intracranial haemorrhage.^12^ Together, these data support the view that intermittently sampled BP variability may better reflect longer-term haemodynamic instability relevant to global functional outcomes, whereas detecting early HT may require higher temporal resolution to capture short-lived BP fluctuations immediately after reperfusion.

The continuous non-invasive monitoring approach used in our study offers a major advantage by enabling minute-level and real-time assessment of BP variability. Blood pressure variation occurring on minute timescales may become particularly deleterious in the setting of impaired cerebral autoregulation, a well-described consequence of acute ischaemia.^18^ While hypertensive peaks may damage the blood–brain barrier and precipitate microvascular leakage,^8,19^ hypotensive drops could further worsen ischaemia in an already vulnerable region.^20^ It is noteworthy that randomised trials investigating intensive BP lowering after EVT^4,6,21,22^ were not designed to detect such short-lived fluctuations. Our work suggest that these short-lived changes carry significant prognostic information and may represent a therapeutic target in the acute phase after reperfusion.

Several limitations deserve mention. First, tolerance to continuous finger-cuff monitoring tended to decrease over time, limiting data completeness over the 24-h recording and contributing to patient exclusions and limited sample size. Because a substantial proportion of patients were excluded for insufficient data, we provided detailed comparisons between included and excluded patients to allow readers to assess potential selection bias. Importantly, excluded patients did not differ from included patients with respect to HT frequency. Second, we used all HT, which include minor haemorrhagic changes that may be clinically silent, rather than symptomatic haemorrhage as the primary endpoint. Even so, recent evidence indicates that even asymptomatic HT after EVT is associated with poorer functional outcomes and increased mortality, supporting the clinical relevance of using any HT as a primary endpoint in this context.^23–25^ Moreover, stratified subgroup analysis based on the extent of HT could not be performed as HT subtypes were not prospectively collected as part of the study design, and complete imaging datasets were not available for all patients at the time of analysis. Third, data on exact timing and class of post-procedural antihypertensive drugs were not sufficiently available to fully parse medication-driven effects on BP variability. Fourth, the accuracy limitations of non-invasive finger-cuff technologies should be acknowledged. Systems such as ClearSight have shown acceptable mean bias compared with invasive arterial pressure in validation studies, but often with wide limits of agreement, particularly in vasoconstricted or haemodynamically unstable patients.^13^ Such measurement uncertainty should be considered when interpreting absolute BP values and derived variability metrics. Nevertheless, our analyses were conducted in a pragmatic stroke unit context with haemodynamically stable patients. Finally, although continuous measures improved risk prediction compared with intermittent monitoring, overall model performance remained modest, indicating that additional clinical and imaging biomarkers should be integrated to enhance accuracy.

To overcome these limitations, combining continuous BP monitoring with intermittent monitoring and non-invasive assessment of cerebral autoregulation using near-infrared spectroscopy^8^ or transcranial Doppler^26^ could inform therapeutic interventions based on personalised thresholds. Moreover, despite increasing evidence linking BP variability to HT, our understanding of how haemodynamic patterns interact with the underlying biological processes remains limited. Future studies should aim to combine high-resolution BP monitoring with biomarkers of blood–brain barrier disruption,^27^ oxidative stress^28^ or neutrophil activation^29^ to better elucidate the mechanistic pathways leading to HT and identify new therapeutic targets.

## Conclusion

In conclusion, continuous non-invasive BP monitoring provides access to short-timescale BP variability that is not captured by intermittent measurements and improves early identification of patients at risk of HT after EVT. While predictive performance remains modest, integrating continuous BP variability metrics into individualised and multimodal post-EVT strategies may guide more effective interventions. Further exploration of short-term BP control could refine post-EVT management, potentially improving patient outcomes.

## Supplementary Material

aakag012_Supplementary_Material_ESJ_Revised

## Data Availability

The data supporting this study are available from the Corresponding Author upon reasonable request.

## Trial registration

BP-METROLOGY: NCT03359434.
