# Supplementary material for: BP-METROLOGY: non-invasive continuous blood pressure monitoring to predict haemorrhagic transformation after endovascular thrombectomy
Source: Eur Stroke J. 2026 Mar 23;11(3):aakag012. doi: 10.1093/esj/aakag012 (PMC13008327; doi:10.1093/esj/aakag012)
Supplement: aakag012_Supplementary_Material_ESJ_Revised [file aakag012_supplementary_material_esj_revised.docx]

**SUPPLEMENTARY MATERIAL**

**Table S1. Comparison of baseline characteristics and clinical information between patients included and excluded from the analysis.**

**Figure S1.** **Univariable logistic regression for association between BP variability features with post-EVT radiologic hemorrhagic transformation.**

**Table S2. Univariable logistic regression for association between BP variability features derived from intermittent monitoring and post-EVT radiologic hemorrhagic transformation.**

**Table S3. Univariable logistic regression for association between BP variability features derived from continuous monitoring and post-EVT radiologic hemorrhagic transformation.**

**Table S4.** **Multivariable logistic regression for association between BP variability features derived from intermittent monitoring and post-EVT radiologic hemorrhagic transformation.**

**Table S5.** **Multivariable logistic regression for association between BP variability features derived from continuous monitoring and post-EVT radiologic hemorrhagic transformation.**

**Figure S2. Multivariable logistic regression for association between 24-hour aggregated BP variability features with post-EVT radiologic hemorrhagic transformation.**

**Table S6. Multivariable logistic regression for association between 24-hour aggregated BP variability features derived from intermittent monitoring and post-EVT radiologic hemorrhagic transformation.**

**Table S7. Multivariable logistic regression for association between 24-hour aggregated BP variability features derived from continuous monitoring and post-EVT radiologic hemorrhagic transformation.**

**Figure S3. Performance of multivariable logistic regression models trained on intermittent or continuous BP dataset (24-hour aggregated data) for the prediction of post-EVT radiologic hemorrhagic transformation.**

**Table S1. Comparison of baseline characteristics and clinical information between patients included and excluded from the analysis.**

|  | **Period 1 (0-8h)** | | | | | **Period 2 (8-16h)** | | | | | **Period 3 (16-24h)** | | | | |
| --- | --- | --- | --- | --- | --- | --- | --- | --- | --- | --- | --- | --- | --- | --- | --- |
|  | **M** | **Overall (n=455)** | **Excluded (n=256)** | **Included (n=199)** | **P** | **M** | **Overall (n=455)** | **Excluded (n=283)** | **Included (n=172)** | **P** | **M** | **Overall (n=455)** | **Excluded (n=347)** | **Included (n=108)** | **P** |
| **Age (years), median [Q1,Q3]** | 0 | 71.0 [59.0,81.0] | 73.0 [61.0,82.0] | 69.0 [55.0,78.0] | <0.001 | 0 | 71.0 [59.0,81.0] | 73.0 [60.0,82.0] | 69.0 [58.0,79.0] | 0.027 | 0 | 71.0 [59.0,81.0] | 72.0 [59.0,81.0] | 71.0 [59.0,80.0] | 0.644 |
| **Sex, No (%)** | 0 |  |  |  |  | 0 |  |  |  |  | 0 |  |  |  |  |
| Female |  | 232 (51) | 137 (54) | 95 (48) | 0.259 | 0 | 232 (51) | 146 (52) | 86 (50) | 0.816 | 0 | 232 (51) | 175 (50) | 57 (53) | 0.752 |
| Male |  | 223 (49) | 119 (46) | 104 (52) |  |  | 223 (49) | 137 (48) | 86 (50) |  |  | 223 (49) | 172 (50) | 51 (47) |  |
| **Hypertension, No (%)** | 0 | 170 (37) | 96 (38) | 74 (37) | 1.000 | 0 | 170 (37) | 106 (37) | 64 (37) | 1.000 | 0 | 170 (37) | 137 (39) | 33 (31) | 0.119 |
| **Diabetes, No (%)** | 0 | 364 (80) | 201 (79) | 163 (82) | 0.436 | 0 | 364 (80) | 224 (79) | 140 (81) | 0.646 | 0 | 364 (80) | 274 (79) | 90 (83) | 0.393 |
| **Smoking, No (%)** | 0 | 376 (83) | 211 (82) | 165 (83) | 0.990 | 0 | 376 (83) | 234 (83) | 142 (83) | 1.000 | 0 | 376 (83) | 284 (82) | 92 (85) | 0.512 |
| **Coronary disease, No (%)** | 0 | 421 (93) | 234 (91) | 187 (94) | 0.394 | 0 | 421 (93) | 259 (92) | 162 (94) | 0.387 | 0 | 421 (93) | 318 (92) | 103 (95) | 0.281 |
| **Prior stroke or TIA, No (%)** | 0 | 389 (85) | 212 (83) | 177 (89) | 0.088 | 0 | 389 (85) | 236 (83) | 153 (89) | 0.135 | 0 | 389 (85) | 296 (85) | 93 (86) | 0.959 |
| **Any antithrombotic treatment, No (%)** | 0 | 266 (58) | 142 (55) | 124 (62) | 0.170 | 0 | 266 (58) | 160 (57) | 106 (62) | 0.332 | 0 | 266 (58) | 205 (59) | 61 (56) | 0.714 |
| **Antiplatelet treatment, No (%)** | 0 | 99 (22) | 60 (23) | 39 (20) | 0.384 | 0 | 99 (22) | 66 (23) | 33 (19) | 0.358 | 0 | 99 (22) | 74 (21) | 25 (23) | 0.789 |
| **Dual antiplatelet treatment, No (%)** | 0 | 4 (1) | 3 (1) | 1 (1) | 0.635 | 0 | 4 (1) | 2 (1) | 2 (1) | 0.635 | 0 | 4 (1) | 3 (1) | 1 (1) | 1.000 |
| **Anticoagulant treatment, No (%)** | 0 | 87 (19) | 50 (20) | 37 (19) | 0.895 | 0 | 87 (19) | 55 (19) | 32 (19) | 0.924 | 0 | 87 (19) | 64 (18) | 23 (21) | 0.604 |
| **Admission MBP (mmHg), median [Q1,Q3]** | 30 | 103 [92,116] | 105 [94,117] | 100 [90,114] | 0.010 | 30 | 103 [92,116] | 104 [94,116] | 100 [91,114] | 0.080 | 30 | 103 [92,116] | 103 [93,116] | 100 [92,112] | 0.086 |
| **NIHSS at admission, median [Q1,Q3]** | 3 | 15.0 [11.0,19.0] | 15.5 [11.0,20.0] | 15.0 [11.0,19.0] | 0.430 | 3 | 15.0 [11.0,19.0] | 15.0 [10.0,20.0] | 15.0 [11.0,19.0] | 0.886 | 3 | 15.0 [11.0,19.0] | 15.0 [10.0,19.0] | 16.0 [13.0,19.0] | 0.096 |
| **NIHSS (categorical), median [Q1,Q3]** | 3 |  |  |  | 0.406 | 3 |  |  |  | 0.700 | 3 |  |  |  | 0.358 |
| 0 to 4 |  | 29 (6) | 12 (5) | 17 (9) |  |  | 29 (6) | 18 (6) | 11 (6) |  |  | 29 (6) | 26 (8) | 3 (3) |  |
| 5 to 15 |  | 207 (46) | 116 (46) | 91 (46) |  |  | 207 (46) | 131 (47) | 76 (44) |  |  | 207 (46) | 157 (46) | 50 (46) |  |
| 16 to 20 |  | 133 (29) | 78 (31) | 55 (28) |  |  | 133 (29) | 77 (28) | 56 (33) |  |  | 133 (29) | 99 (29) | 34 (31) |  |
| ≥ 21 |  | 83 (18) | 47 (19) | 36 (18) |  |  | 83 (18) | 54 (19) | 29 (17) |  |  | 83 (18) | 62 (18) | 21 (19) |  |
| **IV thrombolysis, No (%)** | 0 | 233 (51) | 124 (48) | 109 (55) | 0.212 | 0 | 233 (51) | 145 (51) | 88 (51) | 1.000 | 0 | 233 (51) | 175 (50) | 58 (54) | 0.629 |

|  | **Period 1 (0-8h)** | | | | | **Period 2 (8-16h)** | | | | | **Period 3 (16-24h)** | | | | |
| --- | --- | --- | --- | --- | --- | --- | --- | --- | --- | --- | --- | --- | --- | --- | --- |
|  | **M** | **Overall (n=455)** | **Excluded (n=245)** | **Included (n=210)** | **P** | **M** | **Overall (n=455)** | **Excluded (n=274)** | **Included (n=181)** | **P** | **M** | **Overall (n=455)** | **Excluded (n=343)** | **Included (n=112)** | **P** |
| **mTICI score, No (%)** | 1 |  |  |  |  | 1 |  |  |  |  | 1 |  |  |  |  |
| 0 |  | 9 (2) | 5 (2) | 4 (2) | 0.389 |  | 9 (2) | 5 (2) | 4 (2) | 0.638 |  | 9 (2) | 5 (1) | 4 (4) | 0.446 |
| 1 |  | 1 (0) | 1 (0) |  |  |  | 1 (0) |  | 1 (1) |  |  | 1 (0) | 1 (0) |  |  |
| 2A |  | 10 (2) | 8 (3) | 2 (1) |  |  | 10 (2) | 7 (2) | 3 (2) |  |  | 10 (2) | 9 (3) | 1 (1) |  |
| 2B |  | 189 (42) | 110 (43) | 79 (40) |  |  | 189 (42) | 121 (43) | 68 (40) |  |  | 189 (42) | 142 (41) | 47 (44) |  |
| 3 |  | 245 (54) | 131 (51) | 114 (57) |  |  | 245 (54) | 149 (53) | 96 (56) |  |  | 245 (54) | 189 (55) | 56 (52) |  |
| **Any periprocedural antithrombotics, No (%)** | 1 | 211 (46) | 127 (50) | 84 (42) | 0.130 | 1 | 211 (46) | 138 (49) | 73 (42) | 0.212 | 1 | 211 (46) | 161 (47) | 50 (46) | 1.000 |
| **Intra-arterial thrombolysis, No (%)** | 0 | 24 (5) | 18 (7) | 6 (3) | 0.091 | 0 | 24 (5) | 17 (6) | 7 (4) | 0.496 | 0 | 24 (5) | 18 (5) | 6 (6) | 1.000 |
| **Periprocedural heparin, No (%)** | 0 | 176 (39) | 104 (41) | 72 (36) | 0.385 | 0 | 176 (39) | 115 (41) | 61 (35) | 0.318 | 0 | 176 (39) | 136 (39) | 40 (37) | 0.773 |
| **Periprocedural antiplatelet, No (%)** | 0 | 24 (5) | 15 (6) | 9 (5) | 0.673 | 0 | 24 (5) | 18 (6) | 6 (3) | 0.266 | 0 | 24 (5) | 18 (5) | 6 (6) | 1.000 |
| **Post-procedural antihypertensive treatment, No (%)** | 0 | 167 (37) | 84 (33) | 83 (42) | 0.064 | 0 | 167 (37) | 94 (33) | 73 (42) | 0.060 | 0 | 167 (37) | 120 (35) | 47 (44) | 0.117 |
| **Hemorrhagic transformation, No (%)** | 36 | 135 (32) | 77 (35) | 58 (29) | 0.240 | 36 | 135 (32) | 81 (33) | 54 (31) | 0.845 | 36 | 135 (32) | 102 (33) | 33 (31) | 0.757 |

Abbreviations: HT, hemorrhagic transformation; M: missing data; MBP: mean blood pressure; NIHSS, National Institutes of Health Stroke Scale; P: p-value; SD: standard-deviation; TIA: transient ischemic attack; TICI, Thrombolysis in Cerebral Infarction.

**Figure S1.** **Univariable logistic regression for association between BP variability features with post-EVT radiologic hemorrhagic transformation.**


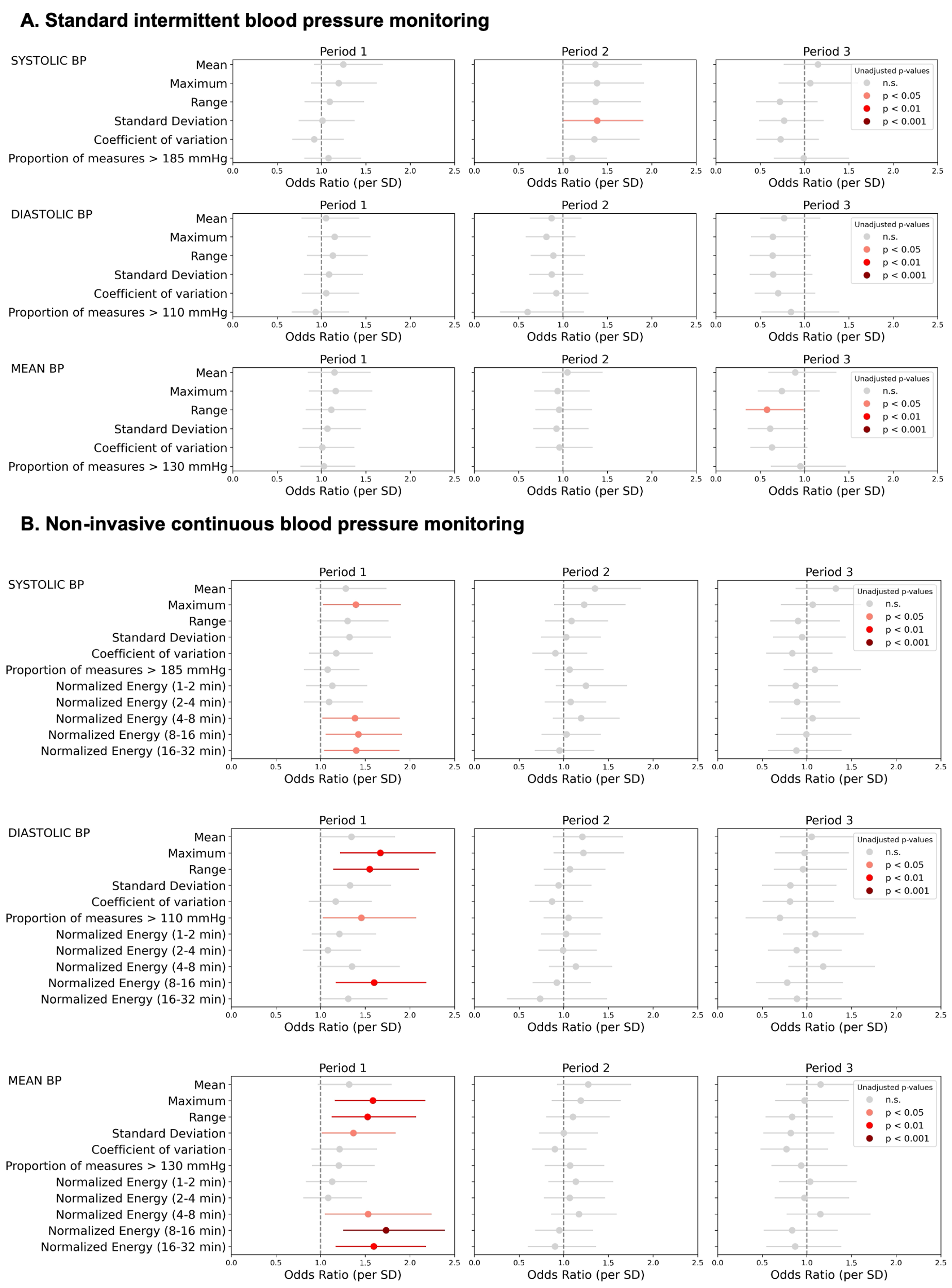


Effect of a one standard deviation (SD) shift on the odds of radiologic hemorrhagic transformation (HT) for each period, for features extracted from intermittent monitoring (A) and noninvasive continuous monitoring (B). Units: mean, maximum, range, and standard deviation in mmHg; coefficient of variation and proportion of measures above an absolute threshold in %; wavelet coefficients normalized energy in mmHg²/min.

**Table S2.** **Univariable logistic regression for association between BP variability features derived from intermittent monitoring and post-EVT radiologic hemorrhagic transformation.**

|  |  | **Period 1 (0-8h)** | | **Period 2 (8-16h)** | | **Period 3 (8-24h)** | |
| --- | --- | --- | --- | --- | --- | --- | --- |
| **Metric** | **Variable** | **Odds Ratio**  **(95% CI)** | **P-value** | **Odds Ratio**  **(95% CI)** | **P-value** | **Odds Ratio**  **(95% CI)** | **P-value** |
| SYSTOLIC | Mean | 1.24 (0.92–1.69) | 0.162 | 1.36 (0.99–1.89) | 0.061 | 1.15 (0.76–1.73) | 0.502 |
|  | Maximum | 1.19 (0.88–1.62) | 0.255 | 1.38 (1.0–1.91) | 0.050 | 1.06 (0.71–1.6) | 0.767 |
|  | Range | 1.09 (0.81–1.48) | 0.565 | 1.37 (0.99–1.88) | 0.054 | 0.72 (0.45–1.15) | 0.167 |
|  | Standard Deviation | 1.01 (0.74–1.37) | 0.945 | 1.38 (1.01–1.91) | 0.046 | 0.77 (0.49–1.22) | 0.261 |
|  | Coefficient of variation | 0.92 (0.67–1.25) | 0.585 | 1.35 (0.98–1.86) | 0.064 | 0.73 (0.46–1.16) | 0.185 |
|  | Proportion of measures > 185 mmHg | 1.08 (0.81–1.45) | 0.608 | 1.1 (0.81–1.5) | 0.526 | 0.99 (0.65–1.5) | 0.964 |
| DIASTOLIC | Mean | 1.05 (0.78–1.43) | 0.746 | 0.87 (0.63–1.21) | 0.409 | 0.77 (0.5–1.18) | 0.227 |
|  | Maximum | 1.15 (0.85–1.55) | 0.374 | 0.81 (0.58–1.14) | 0.229 | 0.64 (0.4–1.04) | 0.074 |
|  | Range | 1.13 (0.84–1.52) | 0.431 | 0.89 (0.63–1.25) | 0.498 | 0.64 (0.38–1.07) | 0.091 |
|  | Standard Deviation | 1.09 (0.8–1.47) | 0.596 | 0.87 (0.62–1.23) | 0.434 | 0.65 (0.38–1.09) | 0.101 |
|  | Coefficient of variation | 1.05 (0.78–1.43) | 0.737 | 0.92 (0.66–1.29) | 0.637 | 0.7 (0.44–1.12) | 0.138 |
|  | Proportion of measures > 110 mmHg | 0.93 (0.67–1.31) | 0.692 | 0.6 (0.29–1.24) | 0.166 | 0.85 (0.51–1.39) | 0.513 |
| MEAN | Mean | 1.15 (0.85–1.55) | 0.379 | 1.05 (0.76–1.45) | 0.769 | 0.9 (0.59–1.36) | 0.603 |
|  | Maximum | 1.16 (0.86–1.57) | 0.335 | 0.94 (0.68–1.3) | 0.700 | 0.74 (0.47–1.17) | 0.200 |
|  | Range | 1.11 (0.82–1.5) | 0.496 | 0.96 (0.69–1.33) | 0.789 | 0.58 (0.34–0.99) | 0.045 |
|  | Standard Deviation | 1.07 (0.79–1.44) | 0.680 | 0.93 (0.66–1.29) | 0.647 | 0.61 (0.36–1.04) | 0.070 |
|  | Coefficient of variation | 1.01 (0.74–1.37) | 0.955 | 0.96 (0.69–1.33) | 0.808 | 0.63 (0.39–1.03) | 0.067 |
|  | Proportion of measures > 130 mmHg | 1.03 (0.76–1.38) | 0.864 | 0.0 (0.0–inf) | 1.000 | 0.95 (0.62–1.47) | 0.821 |

Effect of a one standard deviation (SD) shift on the odds of radiologic hemorrhagic transformation (HT) for each period for features derived from intermittent monitoring. Units: mean, maximum, range, and standard deviation in mmHg; coefficient of variation and proportion of measures above an absolute threshold in %; wavelet coefficients normalized energy in mmHg²/min. NA indicates non-estimable OR due to sparse or invariant predictor values that precluded model convergence.

**Table S3.** **Univariable logistic regression for association between BP variability features derived from continuous monitoring and post-EVT radiologic hemorrhagic transformation.**

|  |  | **Period 1 (0-8h)** | | **Period 2 (8-16h)** | | **Period 3 (8-24h)** | |
| --- | --- | --- | --- | --- | --- | --- | --- |
| **Metric** | **Variable** | **Odds Ratio (95% CI)** | **P value** | **Odds Ratio (95% CI)** | **P value** | **Odds Ratio (95% CI)** | **P value** |
| SYSTOLIC | Mean | 1.28 (0.94–1.74) | 0.113 | 1.35 (0.98–1.86) | 0.069 | 1.32 (0.87–2.0) | 0.185 |
|  | Maximum | 1.4 (1.03–1.9) | **0.034** | 1.23 (0.89–1.69) | 0.208 | 1.07 (0.71–1.6) | 0.759 |
|  | Range | 1.3 (0.96–1.76) | 0.086 | 1.09 (0.79–1.5) | 0.609 | 0.9 (0.59–1.37) | 0.621 |
|  | Standard Deviation | 1.32 (0.98–1.79) | 0.066 | 1.03 (0.74–1.42) | 0.871 | 0.95 (0.62–1.44) | 0.795 |
|  | Coefficient of variation | 1.17 (0.87–1.58) | 0.297 | 0.91 (0.65–1.26) | 0.559 | 0.84 (0.54–1.29) | 0.418 |
|  | Proportion of measures > 185 mmHg | 1.08 (0.81–1.44) | 0.599 | 1.06 (0.78–1.45) | 0.687 | 1.09 (0.74–1.6) | 0.668 |
|  | Normalized Energy (1-2 min) | 1.13 (0.84–1.52) | 0.422 | 1.25 (0.91–1.71) | 0.167 | 0.87 (0.57–1.35) | 0.544 |
|  | Normalized Energy (2-4 min) | 1.09 (0.81–1.48) | 0.552 | 1.08 (0.79–1.48) | 0.645 | 0.89 (0.58–1.38) | 0.603 |
|  | Normalized Energy (4-8 min) | 1.39 (1.02–1.88) | **0.038** | 1.19 (0.88–1.63) | 0.263 | 1.06 (0.71–1.59) | 0.769 |
|  | Normalized Energy (8-16 min) | 1.42 (1.06–1.91) | **0.020** | 1.03 (0.75–1.42) | 0.851 | 0.99 (0.66–1.5) | 0.975 |
|  | Normalized Energy (16-32 min) | 1.4 (1.04–1.88) | **0.027** | 0.95 (0.68–1.34) | 0.781 | 0.88 (0.56–1.39) | 0.590 |
| DIASTOLIC | Mean | 1.34 (0.99–1.83) | 0.062 | 1.21 (0.88–1.66) | 0.245 | 1.05 (0.7–1.59) | 0.803 |
|  | Maximum | 1.67 (1.22–2.29) | **0.001** | 1.22 (0.89–1.68) | 0.224 | 0.97 (0.64–1.47) | 0.896 |
|  | Range | 1.55 (1.14–2.1) | **0.005** | 1.07 (0.78–1.47) | 0.686 | 0.96 (0.63–1.45) | 0.829 |
|  | Standard Deviation | 1.33 (0.99–1.79) | 0.061 | 0.94 (0.68–1.31) | 0.722 | 0.81 (0.5–1.33) | 0.414 |
|  | Coefficient of variation | 1.17 (0.87–1.57) | 0.305 | 0.87 (0.62–1.22) | 0.411 | 0.81 (0.51–1.3) | 0.386 |
|  | Proportion of measures > 110 mmHg | 1.46 (1.03–2.07) | **0.036** | 1.05 (0.77–1.43) | 0.741 | 0.7 (0.31–1.55) | 0.376 |
|  | Normalized Energy (1-2 min) | 1.21 (0.9–1.62) | 0.205 | 1.03 (0.74–1.41) | 0.876 | 1.1 (0.73–1.63) | 0.656 |
|  | Normalized Energy (2-4 min) | 1.08 (0.8–1.46) | 0.610 | 0.99 (0.72–1.37) | 0.959 | 0.88 (0.56–1.39) | 0.593 |
|  | Normalized Energy (4-8 min) | 1.35 (0.97–1.89) | 0.079 | 1.13 (0.83–1.54) | 0.424 | 1.18 (0.79–1.76) | 0.411 |
|  | Normalized Energy (8-16 min) | 1.6 (1.17–2.18) | **0.003** | 0.92 (0.65–1.3) | 0.643 | 0.78 (0.43–1.4) | 0.404 |
|  | Normalized Energy (16-32 min) | 1.31 (0.98–1.75) | 0.071 | 0.74 (0.36–1.49) | 0.394 | 0.89 (0.57–1.39) | 0.606 |
| MEAN | Mean | 1.32 (0.97–1.8) | 0.079 | 1.27 (0.92–1.75) | 0.141 | 1.15 (0.76–1.74) | 0.496 |
|  | Maximum | 1.59 (1.16–2.17) | **0.004** | 1.19 (0.86–1.64) | 0.285 | 0.97 (0.64–1.47) | 0.898 |
|  | Range | 1.52 (1.12–2.07) | **0.007** | 1.1 (0.8–1.52) | 0.544 | 0.83 (0.54–1.29) | 0.414 |
|  | Standard Deviation | 1.37 (1.01–1.84) | **0.041** | 1.0 (0.72–1.38) | 0.997 | 0.82 (0.51–1.31) | 0.403 |
|  | Coefficient of variation | 1.21 (0.9–1.63) | 0.209 | 0.9 (0.64–1.26) | 0.536 | 0.77 (0.48–1.24) | 0.283 |
|  | Proportion of measures > 130 mmHg | 1.2 (0.9–1.61) | 0.205 | 1.07 (0.79–1.45) | 0.657 | 0.94 (0.6–1.46) | 0.768 |
|  | Normalized Energy (1-2 min) | 1.13 (0.84–1.52) | 0.430 | 1.13 (0.83–1.55) | 0.432 | 1.03 (0.69–1.56) | 0.871 |
|  | Normalized Energy (2-4 min) | 1.09 (0.8–1.46) | 0.592 | 1.07 (0.78–1.46) | 0.689 | 0.97 (0.64–1.47) | 0.890 |
|  | Normalized Energy (4-8 min) | 1.53 (1.05–2.24) | **0.028** | 1.17 (0.86–1.59) | 0.316 | 1.15 (0.77–1.71) | 0.490 |
|  | Normalized Energy (8-16 min) | 1.73 (1.25–2.39) | **0.001** | 0.95 (0.68–1.33) | 0.766 | 0.83 (0.52–1.35) | 0.459 |
|  | Normalized Energy (16-32 min) | 1.6 (1.17–2.18) | **0.003** | 0.9 (0.6–1.36) | 0.624 | 0.87 (0.55–1.38) | 0.555 |

Effect of a one standard deviation (SD) shift on the odds of radiologic hemorrhagic transformation (HT) for each period for features derived from noninvasive continuous monitoring. Units: mean, maximum, range, and standard deviation in mmHg; coefficient of variation and proportion of measures above an absolute threshold in %; wavelet coefficients normalized energy in mmHg²/min.

**Table S4.** **Multivariable logistic regression for association between BP variability features derived from intermittent monitoring and post-EVT radiologic hemorrhagic transformation.**

|  |  | **Period 1 (0-8h)** | | **Period 2 (8-16h)** | | **Period 3 (8-24h)** | |
| --- | --- | --- | --- | --- | --- | --- | --- |
| **Metric** | **Variable** | **Odds Ratio**  **(95% CI)** | **P value** | **Odds Ratio**  **(95% CI)** | **P value** | **Odds Ratio**  **(95% CI)** | **P value** |
| SYSTOLIC | Mean | 1.22 (0.83–1.78) | 0.314 | 1.21 (0.82–1.79) | 0.326 | 1.14 (0.69–1.88) | 0.605 |
|  | Maximum | 1.24 (0.86–1.79) | 0.249 | 1.23 (0.83–1.82) | 0.308 | 1.08 (0.66–1.77) | 0.756 |
|  | Range | 1.14 (0.82–1.58) | 0.435 | 1.3 (0.91–1.85) | 0.148 | 0.8 (0.49–1.31) | 0.373 |
|  | Standard Deviation | 1.02 (0.73–1.42) | 0.906 | 1.34 (0.94–1.92) | 0.105 | 0.83 (0.51–1.36) | 0.464 |
|  | Coefficient of variation | 0.95 (0.69–1.32) | 0.780 | 1.35 (0.95–1.92) | 0.091 | 0.81 (0.49–1.32) | 0.398 |
|  | Proportion of measures > 185 mmHg | 1.12 (0.81–1.55) | 0.487 | 0.99 (0.72–1.37) | 0.975 | 0.84 (0.37–1.93) | 0.686 |
| DIASTOLIC | Mean | 0.97 (0.65–1.43) | 0.859 | 0.74 (0.5–1.09) | 0.123 | 0.75 (0.46–1.23) | 0.257 |
|  | Maximum | 1.13 (0.81–1.6) | 0.470 | 0.71 (0.48–1.05) | 0.090 | 0.59 (0.32–1.07) | 0.080 |
|  | Range | 1.13 (0.83–1.54) | 0.429 | 0.86 (0.59–1.24) | 0.408 | 0.57 (0.29–1.11) | 0.099 |
|  | Standard Deviation | 1.09 (0.8–1.49) | 0.580 | 0.83 (0.57–1.21) | 0.330 | 0.54 (0.27–1.07) | 0.079 |
|  | Coefficient of variation | 1.1 (0.8–1.5) | 0.558 | 0.92 (0.64–1.32) | 0.658 | 0.67 (0.38–1.17) | 0.161 |
|  | Proportion of measures > 110 mmHg | 0.92 (0.63–1.36) | 0.685 | 0.59 (0.29–1.21) | 0.148 | 0.32 (0.05–1.88) | 0.206 |
| MEAN | Mean | 1.08 (0.72–1.63) | 0.697 | 0.88 (0.6–1.3) | 0.517 | 0.86 (0.52–1.43) | 0.564 |
|  | Maximum | 1.15 (0.8–1.64) | 0.448 | 0.79 (0.53–1.17) | 0.241 | 0.71 (0.41–1.23) | 0.219 |
|  | Range | 1.12 (0.82–1.54) | 0.463 | 0.92 (0.64–1.32) | 0.654 | 0.56 (0.29–1.06) | 0.076 |
|  | Standard Deviation | 1.07 (0.78–1.47) | 0.661 | 0.88 (0.61–1.27) | 0.492 | 0.59 (0.32–1.1) | 0.097 |
|  | Coefficient of variation | 1.06 (0.77–1.45) | 0.739 | 0.96 (0.68–1.37) | 0.834 | 0.66 (0.38–1.12) | 0.124 |
|  | Proportion of measures > 130 mmHg | 1.02 (0.73–1.42) | 0.919 | 0.0 (0.0–inf) | 1.000 | 0.42 (0.07–2.33) | 0.318 |

Effect of a one standard deviation (SD) shift on the odds of radiologic hemorrhagic transformation (HT) for each period, for features derived from intermittent monitoring. Models were adjusted for admission NIHSS, time to reperfusion, intravenous thrombolysis, and use of periprocedural antithrombotic. Units: mean, maximum, range, and standard deviation in mmHg; coefficient of variation and proportion of measures above an absolute threshold in %; wavelet coefficients normalized energy in mmHg²/min. NA indicates non-estimable OR due to sparse or invariant predictor values that precluded model convergence.

**Table S5.** **Multivariable logistic regression for association between BP variability features derived from continuous monitoring and post-EVT radiologic hemorrhagic transformation.**

|  |  | **Period 1 (0-8h)** | | **Period 2 (8-16h)** | | **Period 3 (8-24h)** | |
| --- | --- | --- | --- | --- | --- | --- | --- |
| **Metric** | **Variable** | **Odds Ratio (95% CI)** | **P value** | **Odds Ratio (95% CI)** | **P value** | **Odds Ratio (95% CI)** | **P value** |
| SYSTOLIC | Mean | 1.28 (0.9–1.83) | 0.172 | 1.25 (0.86–1.81) | 0.243 | 1.49 (0.88–2.5) | 0.137 |
|  | Maximum | 1.43 (1.01–2.04) | **0.044** | 1.13 (0.79–1.61) | 0.515 | 1.09 (0.67–1.76) | 0.726 |
|  | Range | 1.35 (0.98–1.87) | 0.068 | 1.02 (0.73–1.44) | 0.893 | 0.88 (0.56–1.4) | 0.602 |
|  | Standard Deviation | 1.33 (0.96–1.84) | 0.081 | 0.96 (0.68–1.35) | 0.799 | 0.9 (0.57–1.42) | 0.643 |
|  | Coefficient of variation | 1.17 (0.86–1.6) | 0.315 | 0.88 (0.62–1.24) | 0.465 | 0.81 (0.51–1.27) | 0.356 |
|  | Proportion of measures > 185 mmHg | 0.97 (0.7–1.34) | 0.850 | 0.9 (0.65–1.26) | 0.540 | 0.88 (0.38–2.05) | 0.774 |
|  | Normalized Energy (1-2 min) | 1.17 (0.85–1.62) | 0.338 | 1.23 (0.87–1.73) | 0.238 | 0.83 (0.52–1.31) | 0.418 |
|  | Normalized Energy (2-4 min) | 1.12 (0.81–1.55) | 0.485 | 1.02 (0.73–1.42) | 0.902 | 0.92 (0.59–1.43) | 0.702 |
|  | Normalized Energy (4-8 min) | 1.35 (0.96–1.9) | 0.085 | 1.1 (0.79–1.54) | 0.565 | 1.06 (0.69–1.64) | 0.779 |
|  | Normalized Energy (8-16 min) | 1.41 (1.04–1.92) | **0.029** | 0.95 (0.67–1.34) | 0.757 | 0.78 (0.44–1.38) | 0.387 |
|  | Normalized Energy (16-32 min) | 1.37 (1.01–1.86) | **0.040** | 0.87 (0.6–1.26) | 0.458 | 0.77 (0.43–1.39) | 0.391 |
| DIASTOLIC | Mean | 1.29 (0.9–1.86) | 0.170 | 1.07 (0.74–1.53) | 0.728 | 1.06 (0.66–1.69) | 0.821 |
|  | Maximum | 1.66 (1.15–2.39) | **0.007** | 1.07 (0.75–1.51) | 0.720 | 0.97 (0.63–1.51) | 0.909 |
|  | Range | 1.51 (1.08–2.11) | **0.015** | 0.99 (0.71–1.38) | 0.941 | 0.97 (0.63–1.48) | 0.874 |
|  | Standard Deviation | 1.29 (0.92–1.79) | 0.135 | 0.89 (0.63–1.26) | 0.511 | 0.82 (0.51–1.33) | 0.424 |
|  | Coefficient of variation | 1.15 (0.83–1.58) | 0.401 | 0.87 (0.61–1.23) | 0.419 | 0.83 (0.52–1.35) | 0.456 |
|  | Proportion of measures > 110 mmHg | 1.3 (0.89–1.9) | 0.177 | 0.95 (0.69–1.31) | 0.763 | 0.44 (0.08–2.51) | 0.354 |
|  | Normalized Energy (1-2 min) | 1.19 (0.87–1.64) | 0.273 | 0.99 (0.7–1.41) | 0.964 | 1.06 (0.7–1.63) | 0.771 |
|  | Normalized Energy (2-4 min) | 1.09 (0.79–1.51) | 0.604 | 0.93 (0.67–1.3) | 0.667 | 0.91 (0.59–1.4) | 0.666 |
|  | Normalized Energy (4-8 min) | 1.35 (0.92–1.97) | 0.126 | 1.01 (0.73–1.4) | 0.945 | 1.18 (0.77–1.82) | 0.450 |
|  | Normalized Energy (8-16 min) | 1.62 (1.16–2.24) | **0.004** | 0.85 (0.6–1.21) | 0.374 | 0.88 (0.51–1.5) | 0.631 |
|  | Normalized Energy (16-32 min) | 1.24 (0.91–1.7) | 0.169 | 0.69 (0.33–1.43) | 0.315 | 0.83 (0.5–1.36) | 0.460 |
| MEAN | Mean | 1.3 (0.9–1.88) | 0.162 | 1.13 (0.78–1.64) | 0.508 | 1.2 (0.73–2.0) | 0.470 |
|  | Maximum | 1.65 (1.14–2.37) | **0.007** | 1.06 (0.74–1.52) | 0.763 | 0.97 (0.61–1.55) | 0.905 |
|  | Range | 1.56 (1.12–2.17) | **0.008** | 1.02 (0.73–1.44) | 0.890 | 0.85 (0.53–1.34) | 0.479 |
|  | Standard Deviation | 1.35 (0.98–1.87) | 0.070 | 0.93 (0.66–1.31) | 0.663 | 0.82 (0.51–1.31) | 0.401 |
|  | Coefficient of variation | 1.2 (0.88–1.64) | 0.249 | 0.88 (0.62–1.25) | 0.479 | 0.79 (0.48–1.27) | 0.326 |
|  | Proportion of measures > 130 mmHg | 1.05 (0.75–1.48) | 0.767 | 0.9 (0.64–1.25) | 0.522 | 0.3 (0.07–1.31) | 0.108 |
|  | Normalized Energy (1-2 min) | 1.14 (0.83–1.57) | 0.430 | 1.1 (0.78–1.55) | 0.590 | 1.03 (0.67–1.61) | 0.879 |
|  | Normalized Energy (2-4 min) | 1.09 (0.79–1.5) | 0.602 | 1.0 (0.72–1.39) | 0.984 | 1.02 (0.67–1.54) | 0.943 |
|  | Normalized Energy (4-8 min) | 1.55 (1.01–2.39) | **0.046** | 1.04 (0.75–1.45) | 0.799 | 1.17 (0.77–1.79) | 0.466 |
|  | Normalized Energy (8-16 min) | 1.75 (1.25–2.46) | **0.001** | 0.86 (0.6–1.23) | 0.414 | 0.82 (0.48–1.38) | 0.453 |
|  | Normalized Energy (16-32 min) | 1.53 (1.11–2.12) | **0.010** | 0.82 (0.51–1.31) | 0.397 | 0.83 (0.49–1.41) | 0.495 |

Effect of a one standard deviation (SD) shift on the odds of radiologic hemorrhagic transformation (HT) for each period for features extracted from noninvasive continuous monitoring. Models were adjusted for admission NIHSS, time to reperfusion, intravenous thrombolysis, and use of periprocedural antithrombotic. Units: mean, maximum, range, and standard deviation in mmHg; coefficient of variation and proportion of measures above an absolute threshold in %; wavelet coefficients normalized energy in mmHg²/min.

**Figure S2. Multivariable logistic regression for association between 24-hour aggregated BP variability features with post-EVT radiologic hemorrhagic transformation.**

**
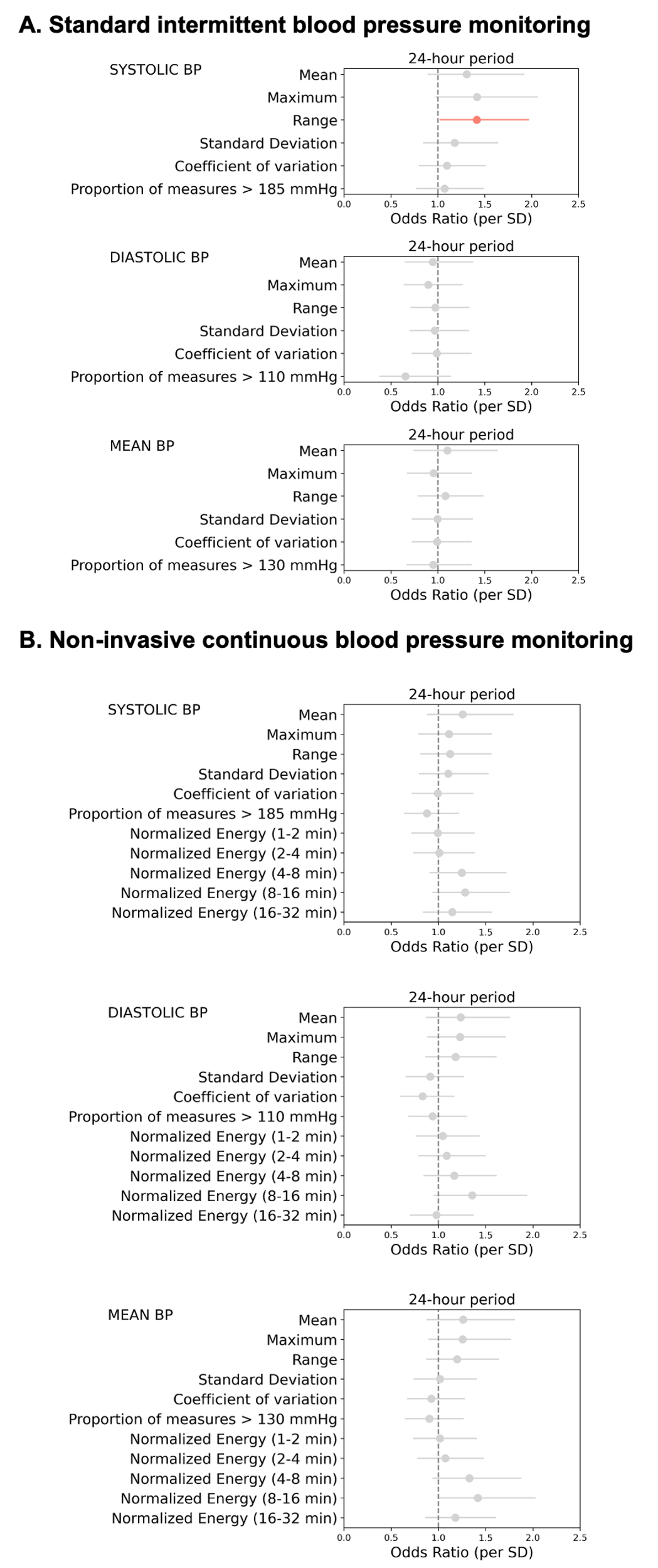
**

Effect of a one standard deviation (SD) shift on the odds of radiologic hemorrhagic transformation (HT) for features extracted from intermittent monitoring (A) and noninvasive continuous monitoring (B), on 24-hour aggregated data. Units: mean, maximum, range, and standard deviation in mmHg; coefficient of variation and proportion of measures above an absolute threshold in %; wavelet coefficients normalized energy in mmHg²/min.

**Table S6. Multivariable logistic regression for association between 24-hour aggregated BP variability features derived from intermittent monitoring and post-EVT radiologic hemorrhagic transformation.**

| **Metric** | **Variable** | **Odds Ratio**  **(95% CI)** | **P value** |
| --- | --- | --- | --- |
| SYSTOLIC | Mean | 1.31 (0.89–1.92) | 0.171 |
|  | Maximum | 1.42 (0.97–2.07) | 0.070 |
|  | Range | 1.41 (1.02–1.97) | 0.040 |
|  | Standard Deviation | 1.18 (0.85–1.64) | 0.332 |
|  | Coefficient of variation | 1.1 (0.8–1.51) | 0.573 |
|  | Proportion of measures > 185 mmHg | 1.07 (0.77–1.49) | 0.686 |
| DIASTOLIC | Mean | 0.94 (0.65–1.38) | 0.768 |
|  | Maximum | 0.9 (0.64–1.27) | 0.540 |
|  | Range | 0.97 (0.71–1.34) | 0.868 |
|  | Standard Deviation | 0.97 (0.7–1.33) | 0.838 |
|  | Coefficient of variation | 0.99 (0.72–1.36) | 0.949 |
|  | Proportion of measures > 110 mmHg | 0.65 (0.38–1.14) | 0.134 |
| MEAN | Mean | 1.1 (0.74–1.64) | 0.638 |
|  | Maximum | 0.96 (0.67–1.37) | 0.803 |
|  | Range | 1.08 (0.79–1.48) | 0.635 |
|  | Standard Deviation | 1.0 (0.72–1.37) | 0.980 |
|  | Coefficient of variation | 0.99 (0.72–1.36) | 0.951 |
|  | Proportion of measures > 130 mmHg | 0.95 (0.66–1.36) | 0.782 |

Effect of a one standard deviation (SD) shift on the odds of radiologic hemorrhagic transformation (HT) for features derived from intermittent data aggregated over the 24-hour recording. Units: mean, maximum, range, and standard deviation in mmHg; coefficient of variation and proportion of measures above an absolute threshold in %; wavelet coefficients normalized energy in mmHg²/min. NA indicates non-estimable OR due to sparse or invariant predictor values that precluded model convergence.

**Table S7. Multivariable logistic regression for association between 24-hour aggregated BP variability features derived from continuous monitoring and post-EVT radiologic hemorrhagic transformation.**

| **Metric** | **Variable** | **Odds Ratio (95% CI)** | **P value** |
| --- | --- | --- | --- |
| SYSTOLIC | Mean | 1.26 (0.88–1.79) | 0.210 |
|  | Maximum | 1.11 (0.79–1.57) | 0.547 |
|  | Range | 1.12 (0.81–1.56) | 0.489 |
|  | Standard Deviation | 1.1 (0.79–1.53) | 0.556 |
|  | Coefficient of variation | 0.99 (0.72–1.37) | 0.967 |
|  | Proportion of measures > 185 mmHg | 0.88 (0.63–1.22) | 0.441 |
|  | Normalized Energy (1-2 min) | 0.99 (0.71–1.39) | 0.972 |
|  | Normalized Energy (2-4 min) | 1.01 (0.73–1.39) | 0.956 |
|  | Normalized Energy (4-8 min) | 1.25 (0.9–1.72) | 0.177 |
|  | Normalized Energy (8-16 min) | 1.28 (0.93–1.76) | 0.123 |
|  | Normalized Energy (16-32 min) | 1.15 (0.84–1.57) | 0.392 |
| DIASTOLIC | Mean | 1.24 (0.87–1.76) | 0.241 |
|  | Maximum | 1.23 (0.88–1.72) | 0.222 |
|  | Range | 1.18 (0.86–1.62) | 0.297 |
|  | Standard Deviation | 0.91 (0.65–1.27) | 0.590 |
|  | Coefficient of variation | 0.83 (0.59–1.17) | 0.291 |
|  | Proportion of measures > 110 mmHg | 0.94 (0.68–1.3) | 0.706 |
|  | Normalized Energy (1-2 min) | 1.05 (0.76–1.44) | 0.786 |
|  | Normalized Energy (2-4 min) | 1.09 (0.79–1.5) | 0.607 |
|  | Normalized Energy (4-8 min) | 1.17 (0.84–1.62) | 0.350 |
|  | Normalized Energy (8-16 min) | 1.36 (0.95–1.94) | 0.093 |
|  | Normalized Energy (16-32 min) | 0.98 (0.7–1.38) | 0.908 |
| MEAN | Mean | 1.26 (0.88–1.81) | 0.211 |
|  | Maximum | 1.26 (0.89–1.77) | 0.187 |
|  | Range | 1.2 (0.87–1.64) | 0.268 |
|  | Standard Deviation | 1.02 (0.74–1.41) | 0.916 |
|  | Coefficient of variation | 0.93 (0.67–1.28) | 0.642 |
|  | Proportion of measures > 130 mmHg | 0.9 (0.64–1.27) | 0.557 |
|  | Normalized Energy (1-2 min) | 1.02 (0.73–1.41) | 0.925 |
|  | Normalized Energy (2-4 min) | 1.07 (0.78–1.48) | 0.669 |
|  | Normalized Energy (4-8 min) | 1.33 (0.94–1.88) | 0.111 |
|  | Normalized Energy (8-16 min) | 1.42 (0.99–2.03) | 0.056 |
|  | Normalized Energy (16-32 min) | 1.18 (0.86–1.61) | 0.305 |

Effect of a one standard deviation (SD) shift on the odds of radiologic hemorrhagic transformation (HT) for features extracted from continuous data aggregated over the 24-hour recording. Models were adjusted for admission NIHSS, time to reperfusion, intravenous thrombolysis, and use of periprocedural antithrombotic. Units: mean, maximum, range, and standard deviation in mmHg; coefficient of variation and proportion of measures above an absolute threshold in %; wavelet coefficients normalized energy in mmHg²/min.

**Figure S3. Performance of multivariable logistic regression models trained on intermittent or continuous BP dataset (24-hour aggregated data) for the prediction of post-EVT radiologic hemorrhagic transformation.**

**
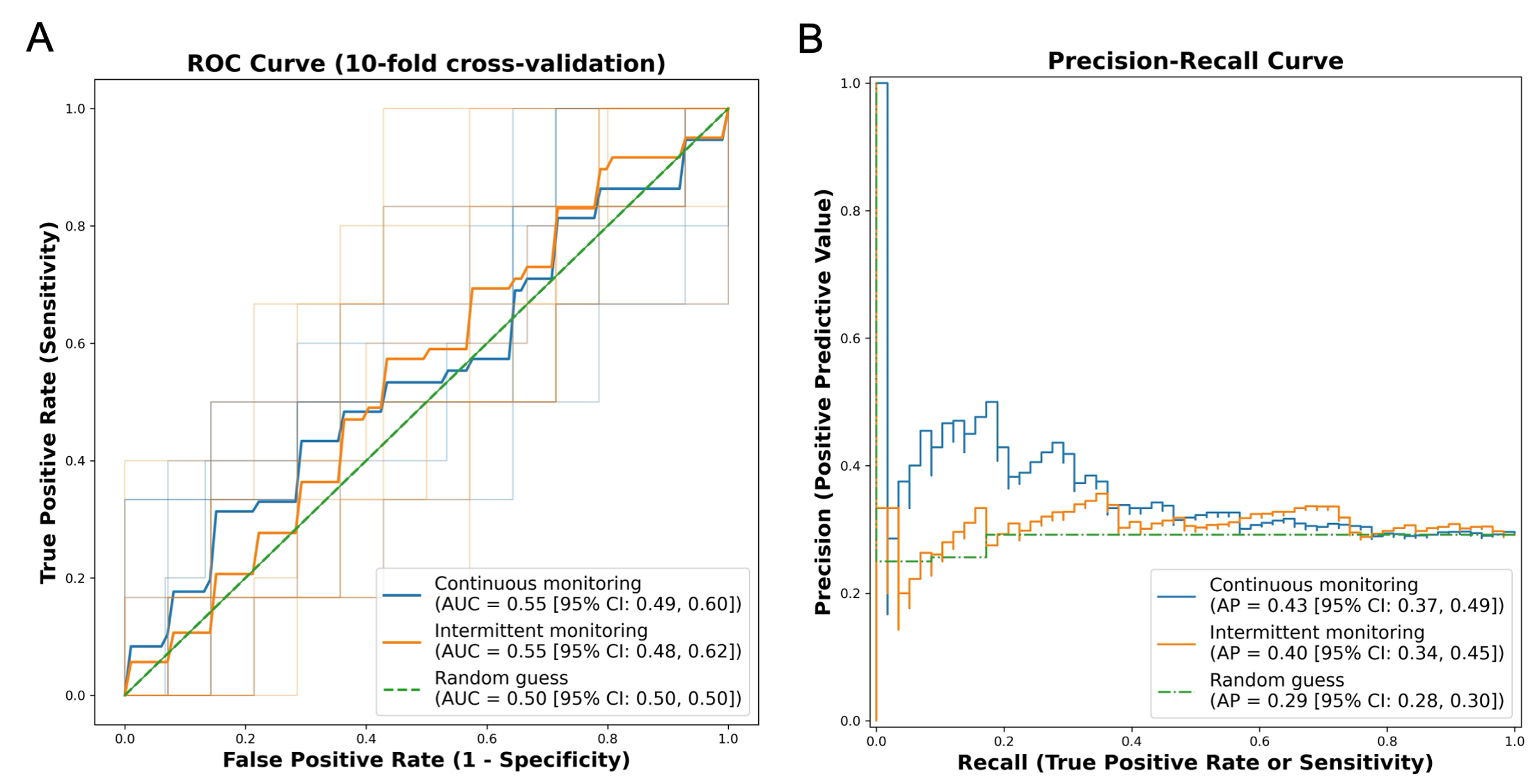
**

Abbreviations: AUC, area under curve; AP, average precision; OR, odds-ratio; ROC, Receiver Operating Characteristic.

ROC curve (A) and precision-recall curve (B) for multivariable logistic regression models trained on BP variability metrics derived from 24-hour aggregated data applying a stratified 10-fold cross-validation.
